# Correction: Clinical Efficacy of ONC201 in H3K27M-Mutant Diffuse Midline Gliomas Is Driven by Disruption of Integrated Metabolic and Epigenetic Pathways

**DOI:** 10.1158/2159-8290.CD-25-0208

**Published:** 2025-03-03

**Authors:** Sriram Venneti, Abed Rahman Kawakibi, Sunjong Ji, Sebastian M. Waszak, Stefan R. Sweha, Mateus Mota, Matthew Pun, Akash Deogharkar, Chan Chung, Rohinton S. Tarapore, Samuel Ramage, Andrew Chi, Patrick Y. Wen, Isabel Arrillaga-Romany, Tracy T. Batchelor, Nicholas A. Butowski, Ashley Sumrall, Nicole Shonka, Rebecca A. Harrison, John de Groot, Minesh Mehta, Matthew D. Hall, Doured Daghistani, Timothy F. Cloughesy, Benjamin M. Ellingson, Kevin Beccaria, Pascale Varlet, Michelle M. Kim, Yoshie Umemura, Hugh Garton, Andrea Franson, Jonathan Schwartz, Rajan Jain, Maureen Kachman, Heidi Baum, Charles F. Burant, Sophie L. Mottl, Rodrigo T. Cartaxo, Vishal John, Dana Messinger, Tingting Qin, Erik Peterson, Peter Sajjakulnukit, Karthik Ravi, Alyssa Waugh, Dustin Walling, Yujie Ding, Ziyun Xia, Anna Schwendeman, Debra Hawes, Fusheng Yang, Alexander R. Judkins, Daniel Wahl, Costas A. Lyssiotis, Daniel de la Nava, Marta M. Alonso, Augustine Eze, Jasper Spitzer, Susanne V. Schmidt, Ryan J. Duchatel, Matthew D. Dun, Jason E. Cain, Li Jiang, Sylwia A. Stopka, Gerard Baquer, Michael S. Regan, Mariella G. Filbin, Nathalie Y.R. Agar, Lili Zhao, Chandan Kumar-Sinha, Rajen Mody, Arul Chinnaiyan, Ryo Kurokawa, Drew Pratt, Viveka N. Yadav, Jacques Grill, Cassie Kline, Sabine Mueller, Adam Resnick, Javad Nazarian, Joshua E. Allen, Yazmin Odia, Sharon L. Gardner, Carl Koschmann

In the original version of this article (1), an error was made during the assembly of the figures. The H3K9me3 immunoblots for DIPG007, DIPGXIII, and H3.3K27M mNSC cells in Fig. 5J are duplicates of the H3K4me3 immunoblots for these cells in the same figure. Figure 5J has been corrected with the original data in the latest online HTML and PDF versions of the article. The authors regret this error.
